# New adult and adolescent tuberculosis vaccines and Indonesia: policy planning and evidence, November 2024

**DOI:** 10.1016/j.vaccine.2025.127490

**Published:** 2025-08-30

**Authors:** Mohammed Alfaqeeh, Sarah Ewart, Rodri Tanoto, Widyaretna Buenastuti, Ina Agustina Isturini, Prima Yosephine, Erlina Burhan, Ria C. Siagian, Sri Rezeki Hadinegoro, Diah Lenggogeni, Richard G. White, Auliya A. Suwantika, Tereza Kasaeva, Birgitte Giersing

**Affiliations:** a Department of Pharmacology and Clinical Pharmacy, Faculty of Pharmacy, Universitas Padjadjaran, Bandung, Indonesia; b Adduna Health Partners, Seattle, Washington, USA; c World Health Organization, Jakarta, Indonesia; d Inke Maris and Associates, Jakarta, Indonesia; e Ministry of Health, Jakarta, Indonesia; f Respiratory Programmatic Implementation and Research Institute, Jakarta, Indonesia,; g Department of Pulmonology and Respiratory Medicine, Faculty of Medicine, Universitas Indonesia, Jakarta, Indonesia; h The Indonesian Food and Drug Authority (BPOM), Jakarta, Indonesia; i Indonesia Technical Advisory Group on Immunization, Jakarta, Indonesia; j Ministry of National Development Planning, Jakarta, Indonesia; k TB Centre, London School of Hygiene & Tropical Medicine, London, United Kingdom of Great Britain and Northern Ireland; l World Health Organization, Geneva, Switzerland

**Keywords:** Tuberculosis, Vaccine, Policy, Indonesia, Evidence

## Abstract

This report summarizes the proceedings of a policy and planning meeting convened in Indonesia, by the Ministry of Health and the World Health Organization, on potential new tuberculosis (TB) vaccines intended for adults and adolescents. Given that Indonesia bears the second highest TB burden globally, and that it is participating in the phase III efficacy study for one of the leading vaccine candidates that could be approved for use by 2028, the meeting aimed to discuss strategies and evidence needs that could accelerate vaccine introduction. The meeting, held in November 2024, was attended by Indonesian policymakers, researchers, health advocates and community representatives, alongside observers from immunization and TB programmes working at the global level, as well as donors. This report offers several key recommendations to ensure new TB vaccines for adults and adolescents are available, accessible and acceptable in Indonesia, as soon as possible after regulatory approval.

The vaccine implementation strategy is expected to be through a stratified, geographical approach, initiating in provinces with the highest disease and socio-economic burden. As such, improved data on disease, infection prevalence and acceptability within different age cohorts and populations are needed to inform national and sub-national (e.g., province or district level) health and economic impact modelling. Early studies on knowledge, attitudes and practices of new TB vaccines for adults and adolescents will help to engage community leaders and advocates and ensure effective, culturally acceptable communication to improve acceptability. Of greatest urgency, a national strategic plan for new TB vaccine introduction, including a roadmap of critical activities, responsible entities and a stakeholder engagement plan are needed.

## 1 Introduction

Tuberculosis (TB) remains a significant public health challenge in Indonesia [1], with the country ranking second in the world for the number of people with TB. [2]. According to the second national TB inventory study conducted in 2023, the TB incidence rate was estimated at 394 per 100,000 population, with a 95 % confidence interval (CI) of 363 to 428 per 100,000 [3]. In line with other high burden countries, the highest burden is in adults and adolescents, who are responsible for the majority of transmission [4].

Indonesia has demonstrated strong ownership in accelerating its response to TB, with clear political will and a commitment by the government to reduce the burden of TB by 50 % by 2029 [5]. Achieving this goal will require innovative solutions, and the introduction of a new, effective TB vaccine for adults and adolescents is considered a critical component of this strategy [6]. The commitment to this ambition was reinforced during a workshop convened the Indonesian Ministry of Health and the World Health Organization (WHO), to guide national efforts to prepare to introduce new TB vaccines. This early preparedness is particularly important considering that Indonesia, classified as an upper middle-income country by 10.13039/100004421the World Bank as of July 2023, is unlikely to be eligible for financing support from 10.13039/100001125Gavi, and will need to secure access to supply and the financial resources to self-procure this new class of vaccines when it becomes available. The meeting, and its remit, received strong support from Indonesia's Minister of Health, Dr. Budi Gunadi Sadikin, who serves as a co-chair of the WHO-led TB Vaccine Accelerator Council [7].

The WHO recently developed a comprehensive framework to guide the preparation for implementation of new TB vaccines for adults and adolescents in high TB burden countries, highlighting the inter-dependency between global enablers and country-specific preparedness initiatives [8]. WHO has also published guidance on the data and evidence needs that are anticipated to be important for global policy consideration [9]. In 2023, WHO established the TB Vaccine Accelerator Council to foster high-level coordination and alignment between funders, global agencies, governments, and end users to expedite vaccine supply and accessibility. In support of the Council, WHO and key stakeholders in the TB community are establishing key technical and strategic working groups, the first of which is focusing on financing and access mechanisms for new TB vaccines [7]. During this meeting, WHO reaffirmed its dedication to supporting Indonesia and other high-burden countries by linking global efforts with national and regional processes and priorities, to ensure the successful integration and rapid uptake of new TB vaccines.

Meeting participants discussed the process and priority evidence needs to accelerate introduction of new TB vaccines in Indonesia, as soon as is feasible after regulatory approval. Specifically, the workshop aimed to:

• Discuss the current TB vaccine pipeline and considerations for potential integration of late-stage candidates into national TB prevention and immunization programs.
• Chart the policy requirements, evidence needs, and critical decisions anticipated to be required in Indonesia to achieve vaccine regulatory approval, policy recommendations, financing, procurement and implementation, as well as the key stakeholders and decision-makers involved.
• Strengthen the foundation for continued dialogue, commitment and integration from national, regional and global stakeholders to accelerate new TB vaccine development and readiness for implementation.

The meeting provided an overview of Indonesia's current TB burden and interventions, highlighting progress in TB prevention and care, involvement in TB vaccine clinical trials, and aspirations for early regulatory approval and local vaccine manufacturing. Discussions also addressed Indonesia's readiness to incorporate new TB vaccines into its national immunization program, considering priority criteria for introduction decision-making. From a global perspective, the sessions emphasized the need to continue multilateral efforts to accelerate TB vaccine development and licensure, produce sufficient supply, and facilitate WHO policy recommendations and financing.

## 2 Late-stage TB vaccine development and global vaccine impact modelling

The TB vaccine pipeline shows considerable promise, with 16 vaccine candidates currently under development, of which 5 are in Phase 3 clinical trials [10]. The recombinant fusion protein M72/AS01E vaccine candidate is the most advanced, having demonstrated 49.7 % (95 % confidence interval [CI], 2.1 to 74.2) efficacy over three years in adults and adolescents in a phase 2b trial [11,12]. A phase 3 trial for M72/AS01E is ongoing across five countries, including Indonesia, and results are expected by 2028 (NCT06062238). Other late-stage candidates include VPM1002, a recombinant BCG vaccine targeting disease prevention (CTRI/2019/01/017026), infection (NCT04351685) and recurrence (NCT03152903). Phase 3 trials for VPM1002 have concluded, and results are pending, as they are for IMMUVAC, a heat-inactivated *Mycobacterium indicus pranii* candidate with a 2-dose regimen (CTRI/2019/01/017026). These candidates, alongside MTBVAC and GamTBVAC, as well as viral vectored and nucleic acid-based candidates represent the current spectrum of vaccine approaches targeting adolescents and adults, the population that has the highest TB burden and likely causes most TB infection transmission [13].

Global TB vaccine impact modelling has provided critical insights into how vaccines could reduce the burden of TB, particularly in high burden, middle-income countries (MICs) like Indonesia. Modelling data suggests that in low- and middle-income countries (LMICs), adolescent and adult vaccination may deliver greater and faster impact than infant vaccination [14]. Vaccines targeting prevention of disease (POD) would yield a faster and greater global impact compared to those targeting prevention of infection (POI) [14,15]. In high-burden countries such as China, South Africa, and India, vaccines that prevent disease in post-infection populations are expected to offer the greatest benefits. Moreover, in some settings, even vaccines with low efficacy such as 20 %, could still be cost-effective when targeted at adolescents and adults. Estimates predicted that introducing vaccines that meet WHO preferred product characteristics [16], such as a POD vaccine with 50 % efficacy and 10 years of protection, could reduce TB incidence by 25 % by 2050, potentially averting 44 million cases and 5 million deaths [17]. The economic modelling for vaccines with these characteristics also highlighted substantial benefits, with every $1 invested in adolescent/adult vaccines potentially yielding $7 in health and economic benefits [18]. This demonstrates the strong economic case for introducing new TB vaccines for this population, not only to reduce disease burden but also to improve health equity, particularly among the poorest populations.

## 3 TB in indonesia

### 3.1 Indonesia's role in TB prevention and care, and the context for TB vaccine development

Indonesia, the most populous nation in Southeast Asia, faces significant challenges in ending TB [19]. As the second-highest TB burden country globally, Indonesia reported 804,836 new cases of people with TB in 2023, with a rising incidence of multi-drug-resistant TB (MDR-TB), exacerbated by pressure on the health system associated with the COVID-19 pandemic [20]. The Indonesian government has committed to ending TB through a multi-sectoral approach involving 15 ministries, aiming for an 80 % reduction in TB incidence and a 90 % reduction in TB-related deaths by 2030, which is emphasized through the current president's quick-win program [21]. The national strategy focuses on strengthening leadership, improving client-centered services, enhancing prevention efforts like TB prevention therapy (TPT), and leveraging research and technological innovations in TB screening, diagnosis, and treatment. However, challenges remain, such as suboptimal screening, limited community involvement, and underutilization of diagnostic services.

Indonesia has positioned itself as a leader in the global fight against TB through collaborative efforts and a strong commitment to advancing TB vaccine research and implementation, actively contributing to the development of new vaccines through its involvement in clinical trials of TB vaccine candidates [22]. Of note is its participation in the ongoing Phase III study for the candidate M72/AS01E (NCT06062238) [23]. This participation not only positions Indonesia as a potential early adopter of this future TB vaccine, but also strengthens Indonesia's position in the global fight against TB and provides critical data to inform both global and national vaccine development and policy processes. Two additional candidates are anticipated to enter clinical trials in Indonesia in 2025–2027. A local manufacturer's recombinant vaccine, presenting 85C and ESAT-6, is anticipated to enter Phase 1. Additionally, CanSino Biologics' inhalable adenoviral-vectored TB vaccine, which presents Ag85A, Mtb32A, and Mtb39A antigens, will enter Phase 1 in collaboration with Etana Biotechnologies, Indonesia. In parallel, the country is strengthening its vaccine manufacturing capacity, with major players like BioFarma, Biotis, and Etana annually producing billions of vaccines, including inactivated, live attenuated virus, and subunit protein/polysaccharide types. Moreover, the establishment of the Indonesia Clinical Research Center (INA-CRC) in October 2024 is expected to further bolster the nation's clinical research infrastructure, facilitating the implementation of new, high-impact trials [24].

Community organizations like Stop TB Indonesia (STPI) are pivotal in combating TB through grassroots advocacy, policy influence, and public awareness campaigns [25]. Their initiatives include securing national commitments, such as a Presidential Regulation on TB, and advocating for village funds to support TB programs. STPI actively engages in global forums like the United Nations High-Level Meeting (UNHLM) on TB and the group of twenty (G20) comprising countries with the world's largest economies, while also fostering community participation at the local level. They have trained youth advocates through the Caraka TB Institute and reached millions via creative media campaigns, including over 300 annual news coverages and substantial social media engagement. These efforts not only promote equitable access to TB prevention and care but also highlight the urgent need for TB vaccine development, leveraging media and government collaboration to drive action.

The meeting agenda highlighted Indonesia's recent engagement in TB vaccine decision-making that used multi-criteria decision analysis (MCDA) in a workshop that took place in 2024 to inform Indonesia's 2025–2029 National Immunization Strategy (NIS). The MCDA workshop utilized the CAPACITI tool [26], and involved relevant key stakeholders in Indonesia, including members of the Indonesia Technical Advisory Group on Immunization (ITAGI). The MCDA exercise identified key criteria to inform decision-making for new vaccine introductions and ranked new TB vaccines for adults and adolescents as the second priority vaccine out of five (after Dengue). A baseline set of criteria from a previous application of CAPACITI for vaccine prioritization in Indonesia (PCV, rotavirus, HPV, and JE) was reviewed for alignment with the current decision question, resulting in the retention of nine criteria. Additionally, key stakeholders were asked to vote on a longlist of 30 pre-defined criteria from the Strategic Multi-Attribute Ranking Tool for Vaccines (SMART Vaccines) to inform the selection process [27]. Through this process, key stakeholders ultimately agreed to add three new criteria—eradication or elimination potential, serious outbreak risk, and equity impact—bringing the total to 12. The final criteria set included disease burden, impact, local production, cold chain needs, vaccine availability, schedule, adverse events following immunization (AEFI), eradication or elimination, outbreak potential, cost-effectiveness, equity impact, and community acceptance. Through consensus discussions, key stakeholders established related indicators, weights, and scoring scales for each criterion. While nine of the 12 criteria were scored and weighted, cost-effectiveness was treated as a dependent variable. At the same time, equity impact and community acceptance were incorporated qualitatively rather than being assigned scores. In particular, key participants critically reviewed the evidence and assessed vaccine options against the established criteria. Using the assigned weights and scoring scales, the final ranking of new vaccine priorities for Indonesia over the next five years was: dengue (246.2) and new TB vaccine (217.5).

New TB vaccines were the only pipeline vaccine included in the MCDA exercise and would have likely ranked higher if more product-related information had been available for the scoring. Six breakout groups were formed based on the decision-making criteria, namely: use case scenarios, health impact modelling, health system readiness, integration with TB interventions, economic impact, and opportunities for local vaccine production.

### 3.2 Indonesia's national immunization strategy (NIS)

Indonesia has a strong commitment to immunization. The national immunization programme (NIP) provides 14 vaccines across various stages of childhood, reaching over 71 million people, including infants, school-age children, women of childbearing age, and high-risk groups [28]. Routine vaccinations like BCG, DPT3, OPV3, and MCV1 have high coverage, while MCV2 and IPV1 coverage remain slightly lower. New vaccines such as HPV, PCV, and rotavirus vaccines have shown promising uptake, with HPV vaccine coverage reaching 49 % in the first three years after introduction in 18 provinces [29]. Factors such as maternal education, household wealth, and the influence of religious leaders, family, and community health workers (cadres) play a significant role in vaccine uptake. However, challenges such as governance issues, high health worker turnover, vaccine shortages, limited financing including other competing health priority programs, data collection gaps due to the transition from manual to digital systems, and vaccine hesitancy remain significant barriers. Best practices for improving immunization uptake include phased vaccine introduction, school-based immunization, advocacy initiatives, and electronic logistics management. The country has also prioritized the use of locally produced vaccines, supporting domestic vaccine manufacturing to enhance accessibility, equity, and sustainability. The new vaccine introduction strategy for 2025–2029 aims to increase coverage, strengthen management, and scale up new vaccines such as the hexavalent vaccine, HPV, and domestically produced PCV and rotavirus vaccines. This plan also includes expanding immunization for Dengue and new TB vaccines. By 2029, the goal is to achieve nationwide coverage with PCV, RV, and HPV vaccines for all eligible children, along with a new TB vaccine demonstration program if a vaccine is available.

### 3.3 TB vaccine impact modelling in Indonesia

Modelling the impact of TB vaccines for adolescents and adults in Indonesia included assessing their effectiveness compared to existing TB prevention and care measures. Modelling typically focuses on evaluating the potential reduction in TB incidence, mortality, and drug-resistant cases, and on cost-effectiveness using the cost per disability-adjusted life year (DALY) averted [30]. While previous studies suggest that TB vaccines in Indonesia could be cost saving or highly cost-effective (presented by R. White during the workshop), the assumptions regarding vaccine efficacy, coverage, and pricing, require further validation in the Indonesian context.

Despite the significant burden of drug-resistant TB in Indonesia, limited modelling has specifically examined the role of TB vaccines in addressing antimicrobial resistance (AMR). While global estimates suggest that AMR could impose a $17.5 trillion economic burden by 2050, further research is needed to quantify the economic and health burden of drug-resistant TB in Indonesia and how vaccines could mitigate these costs [31]. Existing TB models in Indonesia have largely focused on treatment and case detection, leaving a gap in vaccine-specific modelling that considers AMR dynamics [32].

Affordability remains a key challenge despite the potential cost-saving benefits of TB vaccines [33]. To date, no comprehensive budget impact analysis has been conducted to determine the financial feasibility of integrating TB vaccines into Indonesia's national immunization program. Given limited budget, stakeholders emphasized the need for such analyses to assess long-term sustainability and funding strategies. Future modelling should explore phased adoption strategies, prioritizing high-burden areas, health system capacity, and regulatory readiness.

## 4 Priority criteria for TB vaccine introduction in Indonesia

The MCDA exercise (see section 3.1) conducted just prior to this expert meeting identified key criteria to inform decision-making for new vaccine introductions. These criteria were the focus of six break-out groups that discussed key considerations for introducing a new TB vaccine in Indonesia, focusing on: i) use case scenarios, ii) health impact modelling, iii) health system implications, iv) integration into TB programs, v) economic impact, and vi) opportunities for local production and knowledge transfer. These discussions were candidate agnostic, and aimed to outline the current assumptions, conditionalities, knowledge gaps and strategies needed to prepare for effective implementation.

### 4.1 Use case scenarios for TB vaccines in Indonesia

A ‘use-case’ defines for whom and how the vaccine is most likely to be used within the immunization programme. It is essential to identify this, to inform the delivery strategy for the vaccine (see section 4.3) and to model health (section 4.3) and health systems impact (section 4.5).

The discussion on use-case focused on the relative merits of prioritizing high-risk populations, as opposed to high-burden geographical regions for adult and adolescent TB vaccine introduction. Indonesia's prior success with geographical implementation of health interventions was emphasized, with stakeholders recommending a stratified geographical approach, starting with supplementary immunization activities (SIAs), supported by a robust communication plan to involve community leaders and encourage participation. A risk-based population approach, such as prioritizing people living with HIV or diabetes, was thought likely to increase social stigma associated with TB, and the approach could be limited by the demographic surveillance data available. Incidence data on comorbidities, migration, and household contacts, such as healthcare workers and clients with autoimmune diseases, were identified as critical for informing risk assessment within a high risk, population-based approach.

Under the geographical approach, over time, mass campaigns should transition to routine school immunization, with the potential to expand to other provinces based on acceptance and coverage. Drawing on experience from Indonesia's COVID-19 rollout, the group suggested establishing an ad-hoc team for province-based implementation. Operational costs could be managed through cross-sector collaboration.

In supply-constrained scenarios, it was recommended to maintain a geographical implementation approach, focusing on completing mass vaccination campaigns in one province before moving to the next. Delivery considerations highlighted the role of *Puskesmas* (Community Health Centers) as operational hubs, supported by outreach through schools, universities, village halls, and markets, as well as using apps and national ID systems for mobilization, reminders, and monitoring. Importantly, the group noted that vaccine origin, whether imported or locally produced, would not alter the priority use cases and delivery strategies, but may be important for acceptability.

### 4.2 Health impact modelling for late-stage vaccines in Indonesia

In this group, stakeholders discussed the inputs and indicators needed to develop a robust health impact model, based on national epidemiological data, to guide the most cost-effective and impactful introduction strategy for late-stage TB vaccines in Indonesia. Such a model would inform the priority use cases and delivery strategies for new TB vaccines for adults and adolescents, across different sub-national contexts.

The primary objectives of the model include stratifying districts by TB burden, understanding the characteristics of populations most affected within those districts, and providing the Ministry of Health with actionable insights for prioritizing vaccine distribution. Key inputs considered for the model were: infection and disease burden by demographic, population size, risk factors (such as comorbidities like HIV and diabetes), and data related to the vaccine prioritization criteria such as those used by ITAGI, based on conversations with the Ministry of Health. Prevalence studies and population surveys were emphasized as crucial tools for refining these model inputs and better understanding TB infection and disease patterns.

Core discussion points included the need to evaluate relative impact of various TB intervention options, such as the integration of TPT alongside vaccination efforts, and whether screen for previous TB infection to exclude IGRA-negative individuals, given the inability to detect efficacy in this group as part of the phase 3 study because of low incidence. Stakeholders highlighted the need to explore the impact of new vaccines on multi-drug-resistant TB (MDR-TB). Additional model inputs discussed data from active TB detection efforts, susceptibility testing and contact tracing. To improve the accuracy of the model, patient pathways, associated costs, and real-world vaccine effectiveness data would also need to be integrated.

While not typically part of a vaccine impact model, public perception surveys to assess acceptability, willingness to pay, and the threshold for affordability of vaccines are important to inform scenarios and assumptions on uptake and therefore impact. Insights from willingness-to-pay studies for other vaccines, such as rotavirus, were suggested to inform the model.

Modelling initiatives, such as the Indian National Modelling Consortium, were discussed as models for large-scale TB vaccination strategies. Other critical considerations included forecasting vaccine demand to ensure adequate supply and assessing how the introduction of new vaccines might influence the dynamics of existing TB treatments and preventive therapies.

### 4.3 Health systems (delivery) implications for TB vaccine introduction in Indonesia

This group discussed potential delivery strategies for the new TB vaccine in Indonesia, considering the use cases identified in **4.1**. For mass campaigns, institution-based approaches were considered, including school-based vaccination for junior high, high schools, colleges, and boarding schools, as well as workplace vaccinations for sectors with shared living spaces like dormitories, prisons, and private sectors such as taxi drivers and online service drivers. Health system-based approaches, such as during required pre-marital screenings and during other well health visits, and via contacts of people with TB, were also explored.

The group highlighted concerns about the healthcare system burden, with an understanding that any vaccine requiring pre-screening might lead to greatly increased costs and healthcare resource need and may increase vaccine hesitancy due to associated stigma. Vaccine presentation options were considered, with a preference for multiple-dose presentations for institution-based immunization and single-dose presentations for community-based immunization settings. Cold chain capacity was also emphasized, with a need for an assessment of vaccine thermostability to ensure the feasibility of widespread distribution. For tracking vaccinations, the use of ASIK, Indonesia's national electronic health records system was proposed, which is integrated into *SatuSehat*, the country's unified health data platform, to streamline health information across systems and facilitate household contact vaccinations. Lastly, the group recommended that vaccination should be recommended, but not be mandatory to avoid increasing vaccine hesitancy.

### 4.4 Future integration of TB vaccines in TB programs in Indonesia

Stakeholders emphasized the importance of collecting both primary and secondary data as a baseline to consider how a new TB vaccine could be delivered, and to be able measure its potential effectiveness and cost-savings post implementation. These data include existing national health statistics, epidemiological studies, healthcare system reports, and global health data that accurately depict the current TB burden in the context of interventions already in place. For example, would populations already undergoing TPT also be included in vaccine programs? Would TPT delivery enable delivery efforts and resources to be synergized, assuming there is no detrimental interference in effectiveness between interventions?

Vaccine acceptability and accessibility emerged as other critical areas for consideration. There was concern about social and behavioral factors, such as stigma and cultural barriers, particularly regarding the vaccine's acceptability among health professionals and communities. The stakeholders stressed the importance of identifying and addressing these challenges upfront. Knowledge, Attitude, and Practice (KAP) studies were referenced as an effective tool for understanding how communities might perceive the vaccine and how best to address potential barriers to acceptance. In terms of accessibility, it was emphasized that careful assessment would be required to ensure the vaccine's affordability and to evaluate whether *Puskesmas* will have the capacity to manage the increased workload, particularly in administering vaccinations, maintaining records, and providing follow-up care for recipients. Factors such as the timeline to local production and a clear understanding of local regulations should also be considered to ensure effective uptake, especially in resource-limited settings. Engaging health professionals early in the process was also emphasized as a key strategy for ensuring smooth vaccine rollout. A comprehensive communication strategy using both traditional and social media was considered necessary to raise public awareness and educate communities about the vaccine.

In general, stakeholders emphasized the importance of conducting implementation science prior to vaccine approval to address pertinent policy related questions. In addition to vaccine safety and efficacy, vaccine impact modelling and operational research studies should be conducted to identify the optimal use case and mode of delivery to target populations considering the number of doses, in the context of other interventions, including in comparison with the existing BCG vaccine (for infants). The group agreed that assessing the vaccine's cost-effectiveness relative to other TB interventions, including other preventative and anticipated therapeutic innovations, would be essential for guiding policymakers. This modelling should also take into account the potential budgetary implications, as introducing a new vaccine could impact the funds available for Indonesia's TB prevention efforts, and other health initiatives.

### 4.5 Economic impact and budget considerations for TB vaccine introduction in Indonesia

The discussion on the economic impact and budgetary considerations for the introduction of a TB vaccine in Indonesia revealed several critical aspects that need careful evaluation to ensure successful implementation. One of the most important determinants is the position of TB within Indonesia's health agenda, where it will remain a top priority for at least the next five years.

Costing data emerged as a key requirement for evaluating the potential economic impact of the TB vaccine. The cost to vaccinate includes the vaccine price, and the overall cost of introducing the vaccine itself, for example logistics, distribution, storage and administration. These costs will include the infrastructure needed, as well as the costs associated with training healthcare workers and integrating the vaccine into the immunization program. The unit price of the vaccine, and how this is related to the anticipated demand and volume for the Indonesian population, will play a significant role in financial planning.

Stakeholders reported that in order to determine the economic feasibility and sustainability of the TB vaccine, budget impact analysis (BIA) will be crucial, given the competing demands for resources in the health sector. The key focus of the budget impact analysis would be to assess the cost-effectiveness of the vaccine, including its health and economic impact at the population level, evaluate its long-term financial implications and path to sustainability, and determine whether it offers a viable alternative or complement to existing TB prevention and care strategies. ITAGI will play a key role in advising the government on whether to approve the vaccine and its optimal use. However, external financing would likely be required to make the TB vaccine affordable for national procurement. This is particularly important as TB treatment is already largely covered by the Indonesian government, but the vaccine may require additional resources beyond the existing budget for TB-related services.

### 4.6 Vaccine production and opportunities for local production and knowledge transfer

Stakeholders in this group emphasized how vaccine acceptability is linked to local vaccine production. Therefore, early technology transfer of new TB vaccines to Indonesia is an important part of the national plan. A phased approach could be considered, beginning with vaccine importation, progressing to local fill-and-finish production, and eventually achieving full technology transfer of the bulk antigen. In addition to increasing the likelihood of vaccine uptake, this gradual process would help build local manufacturing capacity and reduce reliance on external sources.

Indonesia's local content regulation (TKDN) aims to ensure that 40 % of vaccine components be sourced locally, however, challenges related to the availability of raw materials in Indonesia mean that most vaccines are currently imported. Participants highlighted the need to diversify supply options of both raw materials and new vaccines, to strengthen future the domestic vaccine supply chain potential and capacitate it to prevent stockouts caused by reliance on a single distributor. The group discussed the importance of calibrating local production capacity based on vaccine demand to avoid overproduction or shortages. While local manufacture was seen as ideal, stakeholders noted that it might not necessarily lead to cheaper vaccines due to associated costs with in-licensing, such as intellectual property (IP) and royalties, and the reduced scale may increase the cost of production.

The government's commitment to TB vaccine initiatives was recognized, with participants agreeing that if local manufacturing is not immediately feasible, alternative procurement strategies, such as purchasing through the UNICEF Supply Division, could be considered. However, these alternative pathways would still require local registration with BPOM (the Indonesian Food and Drug Authority) and readiness of local distribution networks. To facilitate early access to an unapproved vaccine, the Special Access Scheme (SAS) could be another pathway while the registration is in process. Additionally, the discussion highlighted that both the public and private sectors have a role to play in ensuring a sustainable vaccine supply strategy. While the public sector currently dominates vaccine production, the private sector could help improve competition and availability in the future.

## 5 Planning for future TB vaccine implementation

The final panel session of the meeting included contributions from five critical stakeholders representing the Indonesian governmental departments on the critical path from regulation to uptake. **Dr. Ria Christine Siagian**, Director of Drug Registration at BPOM; **Prof. Dr. Sri Rezeki Hadinegoro**, Head Director of the Indonesia Technical Advisory Group on Immunization (*ITAGI*); **Prof. Erlina Burhan**, Chair of the TB Expert Committee; **Diah Lenggogeni**, Director of Public Health and Nutrition at National Development Planning Agency (Bappenas); and **Dr. Ina Agustina Isturini**, Director of Communicable Diseases at the Ministry of Health, each provided invaluable insights into the planning and implementation of a future TB vaccine, in alignment with the President's TB elimination goals. The panel discussed various actions that are needed to expedite the pathway to introduction, including the following: quantifying the TB vaccine market in Indonesia, advancing accelerated regulatory pathways, ensuring health system readiness for deployment, preparing for local manufacturing, and engaging with the TB vaccine advocacy community.

### 5.1 Exploring the TB vaccine market and generating demand

Clarity on the expected demand for new TB vaccines for adults and adolescents was identified as a critical factor in understanding the potential volume of doses to negotiate supply and price. The need for robust data on TB infection and disease burden, especially local incidence and prevalence, and vaccine acceptability, was emphasized to accurately gauge the scale of the potential vaccine market and the anticipated vaccination effort. **Prof. Erlina Burhan** emphasized the need for comprehensive data collection, including epidemiological and pharmacoeconomic data, to predict the vaccine's impact. Additionally, she emphasized to start an early educational campaign on new TB vaccines to enhance their acceptability. Both **Dr. Ina Agustina Isturini** and **Prof. Sri Rezeki Hadinegoro** highlighted how this information would inform and enhance vaccine acceptance and create demand across different regions in Indonesia, particularly given that the target population will focus on adolescents and adults.

### 5.2 Assessing health system readiness

The readiness of Indonesia's health system to integrate new TB vaccines for adults and adolescents was discussed in depth, particularly in terms of required infrastructure and human resources. **Dr. Ina Agustina Isturini** highlighted the Ministry of Health's preparation to implement the TB vaccine by 2029, leveraging lessons learned from the COVID-19 vaccination campaign. The health system must ensure adequate human resources, regulatory frameworks, and logistical support, including cold chain distribution. Furthermore, **Diah Lenggogeni** from Bappenas mentioned the importance of multisector coordination for successful implementation, which involves various stakeholders from health, public works, and education sectors to ensure TB elimination is prioritized at all levels.

### 5.3 Pathway from vaccine approval to implementation functions

The regulatory and approval process was addressed by **Dr. Ria Christine Siagian**, who outlined the necessary steps for vaccine approval through BPOM. While initial clinical trials may focus on adolescents and young adults in multiple countries, local data will be crucial to understanding the vaccine's effectiveness and safety profiling in different subgroups. BPOM does not require local clinical trial data for approval, since data from countries or regions with similar genetic backgrounds may be acceptable.

Expedited approval processes for urgent vaccines were discussed, with 100 working days as a maximum for urgent vaccines (widely used and lifesaving) and with the possibility of shortening to as little as 50 working days for a first Investigational New Drug with investment in Indonesia. In practical terms, BPOM would accelerate approval of vaccines for urgent need. After approval, the vaccine recommendations will be considered by ITAGI and vaccine distribution will be handled by the Ministry of Health, in coordination between various stakeholders.

### 5.4 Preparing for local TB vaccine manufacture

The need for Indonesia to develop local manufacturing capabilities for the TB vaccine was highlighted, as being critical to ensure vaccine acceptability in the long-term and a sustainable implementation program. Several potential partnerships are being explored with TB vaccine developers, with the aim to enable technology transfer to ensure the strengthening of production infrastructure. BPOM provides scientific and regulatory advice to vaccine developers and ensures that rigorous scientific and regulatory processes are followed by those who pursue the development of vaccines.

### 5.5 Consulting the TB vaccine advocacy community and priority populations

The engagement of the TB vaccine advocacy community was discussed by multiple panelists. **Prof. Sri Rezeki Hadinegoro** emphasized the importance of involving community stakeholders in vaccine development and introduction, with a focus on clear communication regarding the vaccine's role in TB elimination. Engaging communities in the decision-making process is essential for fostering public trust and acceptance. **Dr. Ina Agustina Isturini** noted that communication strategies must be adapted to the specific challenges of conveying the importance of the TB vaccine, particularly as it may not be as immediately visible as the COVID-19 vaccine.

### 5.6 Engaging community advisory board (CAB) networks

The panel emphasized the importance of involving community advisory boards and networks in the vaccine rollout process. Engaging local populations in discussions about the vaccine's potential benefits and the logistics of its distribution is essential for ensuring broad community support. Both **Prof. Hadinegoro** and **Diah Lenggogeni** highlighted the need for targeted communication strategies that involve community leaders and groups, particularly those in TB hotspots or high-risk environments, to ensure effective vaccine uptake.

## 6 Recommendations

The priority recommendations that emerged from discussions fell into the categories of Availability, Accessibility and Acceptability (see Table 1):

**Table 1:** Workshop recommendations categorized according to the WHO Framework of activities to support Availability, Accessibility and Acceptability. MoH: Ministry of Health; ITAGI: Indonesia Technical Advisory Group for Immunization; MoF: Ministry of Finance; BPOM: The Indonesian Food and Drug Authority. Recommendations are near-term (should be undertaken in parallel to efficacy studies to accelerate policy decision-making), or mid-term (could be undertaken once efficacy data are available and in parallel to preparing for regulatory approval, i.e., soonest 2028).

No.	Recommendation	Stakeholders engaged	Dependencies	Timeframe
1	Availability			
1.1	Develop a national strategic plan, including the National Immunization Strategy, with anticipated phased introduction strategy and scale up of TB vaccine regimens for adults and adolescents which would likely assume i) the vaccine will be imported in the first instance, with the goal of local manufacturing within 5 years of regulatory approval, and ii) a stratified, geographical approach for vaccine implementation through a multi-age cohort campaign, initiating in provinces with highest disease and socio-economic burden.	MoH, ITAGI, WHO Indonesia, Unicef, Gavi, UNDP, academic groups		Near-term
1.2	Revise the National Action Plan for TB Elimination in line with the immunization strategy described in the national strategic plan.	MoH	1.1	Mid-term
1.3	Generate a national demand forecast for the anticipated procurement of the new TB vaccine for Indonesia, aligned with the introduction strategy, and communicate the supply requirements to manufacturers and global partners⁎. This work will inform the recently established TB Vaccine Accelerator's Finance and Access Working Group [34], which is developing a global TB vaccine demand forecast to inform potential novel financing strategies for all countries.	MoH, academic groups, civil society	1.1, 1.5	Near-term, with updates as needed
1.4	Promote robust, affordable and timely supply of new TB vaccines by urgently investing in and facilitating partnerships with vaccine manufacturers in Indonesia, to enable technology transfer of new TB vaccines and manufacturing scale up for implementation.	MoH, academic groups, civil society	1.1, 1.3	Near-term
1.5	Collate or generate improved data on the disease and infection prevalence and risk of transmission within different age cohorts and populations such the general population, household contacts, people living with HIV and diabetics, to improve health and economic modelling estimates.	MoH, academic groups	1.1, 1.2	Near-term
1.6	Develop a Gantt chart (project plan with inter-dependencies, resource allocation and key stakeholders) that maps the critical path of activities across governmental departments, from clinical development, through regulatory approval and policy to implementation. Ensure early and frequent engagement with BPOM, the TB expert committee and ITAGI to discuss data requirements and timelines to initial approval, and to develop a strategy to transition to a locally manufactured vaccine.	MoH, BPOM, Ministry of National Development Planning (Bappenas), ITAGI, WHO Indonesia	1.1, 1.2, 1.3, 1.4, 1.5, 2.1	Near-term
2	Accessibility			
2.1	Develop national and sub-national (e.g., province or district level) health and economic impact models to identity most impactful and cost-effective implementation strategies for new TB vaccines, including scenarios modelling the use of TPT, the inclusion of IGRA-negative populations, and impact on multi-drug-resistant TB.	MoH, academic groups	1.1,1.5	Near-term
2.2	Conduct cost-effectiveness modelling and budget impact studies supported by sub-national disease and health systems data, to explore affordability implications.	MoH, academic groups	1.1, 2.1, 1.5	Near-term
2.3	Plan to identify and include resources for post-marketing surveillance to assess the pharmacovigilance of the vaccine.	MoH	1.5	Mid-term
2.4	Determine the cost-benefit analysis of new TB vaccines, evaluating the implementation costs and the potential reduction in treatment expenses, including those associated with antimicrobial-resistant strains, in both the short and long term after their introduction.	MoH, MoF, academic groups, Bappenas	1.1, 1.5	Mid-term
2.5	Begin engagement with the Bappenas and Ministry of Finance (MoF) to assess potential budget impact in the context of the national strategic plan, considering the proposed use cases, and with the TB Vaccine Accelerator's Finance and Access Working Group to assess the need and options for catalytic external financial assistance, ahead of vaccine availability.	Bappenas, MoH, MoF, WHO	1.1, 1.5, 2.1, 2.2, 2.4.	Near-term
3	Acceptability			
3.1	Develop a national stakeholder engagement plan to proactively engage with all relevant stakeholders from product development to introduction, in particular community leaders and patient advocates, to ensure effective, culturally sensitive communication on TB vaccines.	MoH, WHO Indonesia	1.6	Near-term
3.2	Conduct knowledge, attitudes and practices assessments on new TB vaccines to assess and avoid use cases that may increase the risk of stigmatization and proactively partner with civil society to ensure acceptance.	MoH, Civil society	1.4, 1.5, 2.1	Near-term
3.3	Engage and partner with the TB Vaccine Accelerator's efforts to co-ordinate and integrate TB vaccine related activities across national, regional and global stakeholders.	MoH, WHO	1.1, 1.3, 1.4, 2.5.	Near-term
3.4	Leverage lessons from the COVID-19 vaccine roll-out by implementing large-scale community education campaigns, involving relevant stakeholders and public figures to enhance trust and acceptance of TB vaccination.	MoH	1.1	Mid-term

⁎ It may be beneficial to refer to existing global forecasts, such as the MM Global Health Consulting (MMGH)-commissioned study by the Wellcome Trust, as a foundation for refinement [35]. Additionally, consider collaborating with organizations such as WHO, Gavi, Clinton Health Access Initiative (CHAI), London School of Hygiene and Tropical Medicine (LSHTM), and the International Aids Vaccine Initiative (IAVI), which are collaborating to develop a demand forecast for high-burden countries.

## 7 Conclusions

Indonesia is taking significant and proactive steps to accelerate the development of the first new TB vaccines in over a century and to prepare for their future implementation, driven by strong political will, robust strategic partnerships, and a genuine commitment to advancing the research and development of TB vaccines that could prevent and dramatically reduce the TB burden in Indonesia.

Despite challenges such as budget constraints and the need to deploy this vaccine to a novel age group, the new government intends to prioritize expansion of the immunization program to include TB because of the public health and economic benefits that this new class of vaccines could bring. However, we as Indonesian stakeholders must, in parallel to clinical trials, explore sustainability strategies, such as negotiating with vaccine manufacturers and pursuing technology transfer to enable local production and to promote acceptability. Demonstrating the vaccines' cost-effectiveness through detailed economic modelling will be crucial, highlighting how long-term savings from reduced TB treatment costs can justify the initial investment in vaccine introduction. Furthermore, pilot projects conducted after regulatory approval can provide valuable effectiveness data in the local context, refining vaccination strategies and better assessing market and supply needs.

Successful accelerated implementation of new TB vaccines for adults and adolescents will depend on multi-sectoral collaboration within and between various governmental ministries including health, public works, and education sectors to prioritize TB elimination at all levels of government. Close partnership with international organizations, will be needed to secure affordable vaccine pricing, and optimize sufficient supply.

## Data statement

The summary, conclusions and recommendations presented in this manuscript are derived, de novo, from consultation with key experts on new TB vaccines who are based in Indonesia, from a stakeholder convening conducted in November 2024. The convening was organised by the World Health Organization and the Indonesian Ministry of Health.

## CRediT authorship contribution statement

**Mohammed Alfaqeeh:** Writing – review & editing, Writing – original draft, Project administration. **Sarah Ewart:** Writing – review & editing, Project administration, Methodology, Conceptualization. **Rodri Tanoto:** Writing – review & editing, Validation, Project administration, Conceptualization. **Widyaretna Buenastuti:** Writing – review & editing, Resources, Project administration, Conceptualization. **Ina Agustina Isturini:** Writing – review & editing, Methodology. **Prima Yosephine:** Writing – review & editing, Methodology. **Erlina Burhan:** Writing – review & editing, Methodology. **Ria C. Siagian:** Writing – review & editing, Methodology. **Sri Rezeki Hadinegoro:** Writing – review & editing, Methodology. **Diah Lenggogeni:** Writing – review & editing, Methodology. **Richard G. White:** Writing – review & editing, Methodology, Conceptualization. **Auliya A. Suwantika:** Writing – review & editing, Methodology. **Tereza Kasaeva:** Writing – review & editing, Methodology, Conceptualization. **Birgitte Giersing:** Writing – review & editing, Writing – original draft, Supervision, Methodology, Funding acquisition, Conceptualization.

## Funding

Funding for this stakeholder meeting was gratefully received from the Gates Foundation, award number INV-077504. RGW is funded by the Wellcome Trust (310728/Z/24/Z, 218261/Z/19/Z), NIH (1R01AI147321–01, G-202303-69963, R-202309-71190), EDTCP (RIA208D-2505B), UK MRC (CCF17–7779 via SET Bloomsbury), ESRC (ES/P008011/1), BMGF (INV-004737, INV-035506), Open Philanthropy/Good Ventures (GV673606227), and the WHO (2020/985800–0).

## Declaration of competing interest

The authors declare that they have no known competing financial interests or personal relationships that could have appeared to influence the work reported in this paper.

## Data Availability

No data was used for the research described in the article.
